# Prescription of Aminoglycosides in 23 French Neonatal Intensive Care Units

**DOI:** 10.3390/antibiotics10111422

**Published:** 2021-11-20

**Authors:** Séverine Martin-Mons, Béatrice Gouyon, Séverine Lorrain, Soumeth Abasse, Cénéric Alexandre, Guillaume Binson, Roselyne Brat, Laurence Caeymaex, Yvan Couringa, Cécile Desbruyeres, Marine Dorsi-Di Meglio, Guillaume Escourrou, Florence Flamein, Olivier Flechelles, Olivier Girard, Elsa Kermorvant-Duchemin, Alexandre Lapillonne, Catherine Lafon, Massimo Di Maio, Gaël Mazeiras, Julien Mourdie, Amélie Moussy-Durandy, Anne-Sophie Pages, Duksha Ramful, Hasinirina Razafimahefa, Jean-Marc Rosenthal, Silvia Iacobelli, Jean-Bernard Gouyon

**Affiliations:** 1 Centre D’Etudes Périnatales de L’Océan Indien (UR 7388), Université de La Réunion, 97410 Saint-Pierre, France; simon.lorrain@univ-reunion.fr (S.L.); silvia.iacobelli@chu-reunion.fr (S.I.); 2 Société LogipremF, 97410 Saint-Pierre, France; beatrice.gouyon@logipren.com; 3 Centre Hospitalier de Mayotte, 97600 Mayotte, France; s.abasse@chmayotte.fr; 4 Centre Hospitalier Universitaire de Caen, 14000 Caen, France; alexandre-ce@chu-caen.fr; 5 Centre Hospitalier Universitaire de Poitiers, 86000 Poitiers, France; guillaume.binson@chu-poitiers.fr; 6 Centre Hospitalier Régional d’Orléans, 45100 Orléans, France; roselyne.brat@chr-orleans.fr; 7 Centre Hospitalier Intercommunal de Créteil, 94000 Créteil, France; laurence.caeymaex@chicreteil.fr; 8 Centre Hospitalier Andrée-Rosemont, 97300 Guyane, France; yvan.couringa@ch-cayenne.fr; 9 Centre Hospitalier Métropole Savoie, 73000 Chambéry, France; cecile.desbruyeres@ch-metropole-savoie.fr; 10 Centre Hospitalier Territorial Gaston-Bourret, 98800 Nouméa, France; marine.dorsi@cht.nc; 11 Centre Hospitalier Intercommunal André Grégoire, 93100 Montreuil, France; guillaume.escourrou@ght-gpne.fr; 12 Centre Hospitalier Universitaire de Lille, 59000 Lille, France; florence.flamein@chru-lille.fr; 13 Centre Hospitalier Universitaire de Fort-de-France, 97200 Martinique, France; olivier.flechelles@chu-martinique.fr; 14 Centre Hospitalier de Saint-Denis, 93200 Saint-Denis, France; olivier.girard@ch-stdenis.fr; 15 French Society of Neonatology, 93200 Ecouen, France; elsa.kermorvant@aphp.fr; 16 APHP Hôpital Necker-Enfants Malades, 75015 Paris, France; alexandre.lapillonne@aphp.fr; 17 Centre Hospitalier d’Arras, 62000 Arras, France; catherine.lafon@gh-artoisternois.fr; 18 Centre Hospitalier Universitaire de Nîmes, 30900 Nîmes, France; massimo.dimaio@chu-nimes.fr; 19 Centre Hospitalier de la Côte Basque, 64100 Bayonne, France; gmazeiras@ch-cotebasque.fr; 20 Hôpital Jacques Monod, Groupe Hospitalier du Havre, 76290 Montvilliers, France; julien.mourdie@ch-havre.fr; 21 Centre Hospitalier Intercommunal Poissy, 78300 Poissy, France; adurandy@chi-poissy-st-germain.fr; 22 Centre Hospitalier Public du Cotentin, 50100 Cherbourg-en-Cotentin, France; as.pages@ch-cotentin.fr; 23 Centre Hospitalier Universitaire-Site Nord, Saint-Denis, 97400 Réunion, France; duksha.ramful@chu-reunion.fr; 24 Centre Hospitalier Sud Francilien, 91100 Corbeil-Essonnes, France; hasinirina.razafimahefa@chsf.fr; 25 Centre Hospitalier Universitaire de Pointe-à-Pitre, 97110 Guadeloupe, France; jean-marc.rosenthal@chu-guadeloupe.fr; 26 Centre Hospitalier Universitaire-Site Sud, Saint-Pierre, 97410 Réunion, France

## Abstract

**Background**: Aminoglycosides are the most prescribed antibiotics in neonatal intensive care units (NICU). Reducing exposure to antibiotics in the NICU is highly desirable, particularly through benchmarking methods. **Methods:** Description of aminoglycosides prescriptions in 23 French NICU using the same computerized system over a 4-year period (2017–2020). A benchmarking program of antibiotics prescription was associated. **Results:** The population included 53,818 patients. Exposition rates to gentamicin and amikacin were 31.7% (*n* = 17,049) and 9.1% (*n* = 4894), respectively. Among neonates exposed to gentamicin, 90.4% of gentamicin and 77.6% of amikacin treatments were started within the 1st week of life. Among neonates exposed to amikacin, 77.6% started amikacin within the 1st week. The average daily dose of gentamicin at first prescription increased over the study period from 3.9 in 2017 to 4.4 mg/kg/d in 2020 (*p* < 0.0001). Conversely, the corresponding amikacin daily doses decreased from 13.0 in 2017 to 12.3 mg/kg/d in 2020 (*p* = 0.001). The time interval between the first 2 doses of gentamicin was mainly distributed in 3 values during the first week of life: 49.4% at 24 h, 26.4% at 36 h, and 22.9% at 48 h. At first amikacin prescription, the time interval was distributed in 4 categories: 48% at 24 h, 4.1% at 30 h, 8.5% at 36 h, and 37.1% at 48 h. As compared to literature guidelines, the rates of overdose and underdose in gentamicin (1.5% and 2.7%) and amikacin (0.3% and 1.0%). They significantly decreased for gentamicin over the study period. In multivariate analysis, the factors significantly associated with GENT overdose were the year of admission, prematurity, length of stay, and duration of the treatment. **Conclusion:** This prescription strategy ensured a low rate of overdose and underdose, and some benefits of the benchmarking program is suggested.

## 1. Introduction

Aminoglycosides are the usual partner for betalactams or glycopeptides when neonates are at risk for severe early or late neonatal infection, especially if Gram-negative bacteria are suspected.

Antibiotics are also the most prescribed medications in neonatal wards (NW) [1,2,3]. Gentamicin (GENT) is the leading aminoglycoside, ranking as the 1st or 2nd most prescribed drug in the neonatal intensive care unit (NICU). Amikacin (AMK) has been reported as the 2nd most prescribed aminoglycoside in France and Europe [1,2,3]. 

Computerized prescribing with cognitive assistance (CPOE/CDS) is generally considered an effective way to secure prescribing in neonatal units. This prescribing aid is all the more justified in the case of neonates, as they represent the most prescribing errors of all patients present in a hospital [4]. Computerization of medication order entry in NICU resulted in a significant decrease in the occurrence and severity of medication errors [1,4,5,6]. These errors are favored by the complexity of prescribing, which is linked to the need to adapt the dosage rapidly to their degree of metabolic immaturity (liver, kidney, distribution volumes). Great precision in prescribing is also required, particularly for off-label drugs whose pharmaceutical forms are unsuitable for neonates.

For a long time, antibiotics prescriptions have been audited in NW. These audits were followed by feedback to NW and then corrective actions and improvement, particularly in NICU. These approaches are grouped under the term “Antibiotics Stewardship Program” (ASP) that was shown by recent studies as an efficient way of reducing antibiotics initiation and/or in treatment duration without additional risks [7,8,9,10,11,12,13,14,15,16,17].

Rajar et al. [7] identified 3 groups of actions as the objective of ASP in NICU: restricting initiation of antibiotics, reducing the duration of antibiotics treatments, and implementing various organizational actions. This quality process has been applied to limit the duration of early antibiotherapy to 48 h in the Scout study [8]. Lu et al. [9] reviewed electronic records of all antibiotic use in the NICU and limited the use of first, second-, and third-line antibiotics to restrict the consumption of broad-spectrum antibiotics. 

We recently showed the feasibility of an ASP enlargement by recording all electronic prescribing data in NICU [18]. We particularly focused this ASP on antibiotic exposure rates over a long period of time (2017–2019) and in a large panel of 23 NICU [1]. The annual diffusion of benchmarking results among the participating centers was associated with a global improvement in antibiotics exposure. However, preterm infants less than 32 weeks gestational age (GA) benefited the least from reduced antibiotic exposure [1]. In addition, the qualitative aspects of antibiotic prescribing (i.e., daily dose, time interval between doses, unit dose, administration route) were not addressed, particularly for aminoglycosides treatments which are known to be at risk of nephro- and ototoxicity [19]. This justified the present study designed to identify in-depth the qualitative characteristics of electronic prescriptions of aminoglycosides in our population cohort extended up to 2020.

## 2. Methods

### 2.1. Study Design

This study is a retrospective analysis of electronic prescriptions of aminoglycosides recorded in a 23 NICU network over the 2017–2020 period. Some information on data recording and management are given in two recent articles about global prescriptions in NICU [18] and antibiotic prescriptions [1] over the 2017–2019 period.

The main objective of this study was to assess aminoglycosides’ daily dose (DD), its variability with GA and its annual changes, inter-NICU variability, and conformity with professional recommendations. Secondarily, changes in unit dose (UD) and time interval (TI) between doses were assessed.

### 2.2. Characteristics of the CPOE/CDS System and Prospectively Recorded Data

The CPOE/CDS system (Logipren software) and data recorded for each prescription have been previously described [1,17,18]. In brief, this system allows medication prescription according to indication, GA, postnatal age, postconceptional age, and body weight at birth and on the day of prescription. All electronic prescriptions are automatically stored in local computer servers, and anonymized (deidentification) data is aggregated monthly in a national server. The authorization has been given by the National Commission for Data Protection and Privacy (DE-2015-099; DE-2017-410) and complies with the most recent French regulation MR-003, which governs research in the health field without the need of obtaining specific consent (*Commission National de l’Informatique et des Libertés*, 2018).

### 2.3. The Key Intervention

A benchmarking program focused on the necessity to reduce and shorten antibiotics in NICU [1]. It annually gave globally and at each NICU the rates of exposure to each antibiotic. Results were discussed, and each neonatal ward was allowed to modify (or not) its antibiotics policy.

### 2.4. Inclusion Criteria

Patients were included if they were admitted between 1 January 2017 to 31 December 2020 in a NICU and if the first prescription was before the 28th day of life. The study was limited to injectable intravenous aminoglycoside prescriptions in the first 28 days of life.

### 2.5. Definitions

The prescription indicates a UD (mg/kg) and a TI before the next dose. The DD (mg/kg/d) is calculated for the purpose of the study and in accordance with previous publications (DD = (UD/TI) × 24) to make the results consistent and comparable across studies or NW.

Aminoglycosides over- and under-daily dosing were established by comparing the dosing parameters to a panel of reference values which are the minimum and maximum values available in representative reference literature (Pediatric and Neonatal Dosage Handbook, BNFc–British National Formulary for children, ANSM–Agence National de Sécurité du Médicament, SFN–Société Française de Néonatalogie). More details on references values are in Supplemental Table S1.

This method of identifying over- and under-daily dosing is derived from ASP applied to NICU [10,11,15,16]. Over- and under-daily dosing is defined according to the highest and lowest daily dosing values calculated from available textbooks and recommendations [20].

The end of an aminoglycoside treatment is defined by the cessation of the prescription of the molecule for at least 3 days. A resumption of the drug beyond this period corresponds to a new treatment.

### 2.6. Statistical Analysis

Results were presented using frequency and proportion for discontinuous variables and using the median and interquartile range (IQR) or mean and standard deviation for continuous variables. Cochran–Armitage tests for trend and ANOVA (or Kruskal-Wallis test) were applied in GA and year comparisons.

A stepwise backward logistic regression was performed to identify factors associated with GENT overdosing after selecting them in univariate analysis (comparison of neonates with and without GENT overdosing using chi-square test or Fisher test for binary variables, and Student’s t-test or Wilcoxon-Mann-Whitney for continuous variables). The validity of the logistic models was verified by means of the Hosmer and Lemeshow global adequacy test and with the study of the discriminating power (area under the curve). Statistical analysis was conducted using SAS^®^ software (Version 9.4, SAS Institute, Cary, NC, USA).

## 3. Results

### 3.1. NICU Characteristics

Seventeen hospitals used the Logipren CPOE/CDS for the entire four-year study period, while an additional six hospitals accessed the BPEN network in the first year of registration. The median value for total admissions per hospital was 2091 neonates (IQR: 1613; 2938. Extreme values: 1185; 4633).

### 3.2. Patients Characteristics

**Figure 1:**
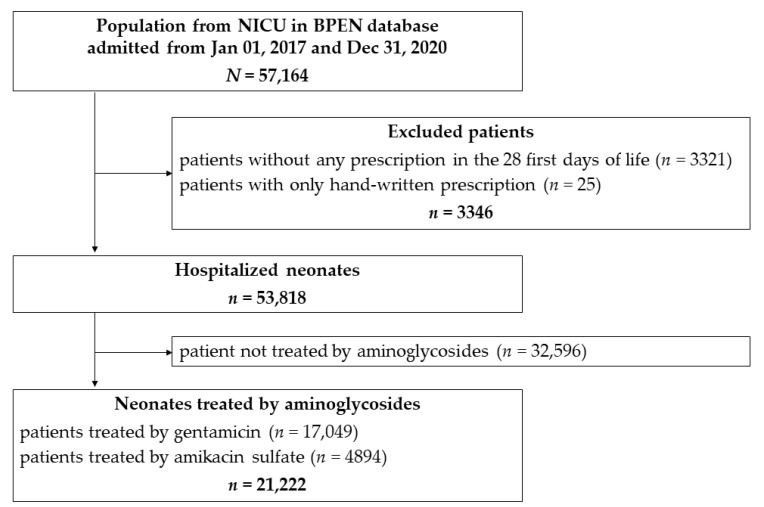
Selection of aminoglycosides treated population.

A total of 53,818 patients were included in the study (Figure 1).

Of these, 54.6% were male, and the median GA was 36.0 weeks (IQR: 33.0; 39.0). The distribution of GA was as follows: 4.1% at 22–26 weeks, 13.4% at 27–31 weeks, 33.7% at 32–36 weeks, and 48.8% at >37 weeks. As for birth weight, the median value was 2480 g (IQR: 1780; 3210). Of the total population, 2.7% had a birth weight of less than 750 g, 4.2% were in the range (750; 999) 10.0% in (1000; 1499) 15.1% in (1500; 1999) and 67.3% above 2000 g. The overall hospital discharge mortality rate was 2.9%. The median length of stay was 8 days (IQR: 3; 21).

### 3.3. Characteristics of Aminoglycosides Prescription

#### 3.3.1. Patient Exposure to Aminoglycosides

Among the 53,818 hospitalized neonates, the overall exposure rate to aminoglycosides was 39.4%, and exposure rates to GENT and AMK were 31.7% and 9.1%, respectively. Of note, 721 patients (1.3%) received both GENT and AMK, with GENT being prescribed first in 86.8% of cases. Marginally, 2 patients received tobramycin.

#### 3.3.2. Exposure to Gentamicin and Amikacin Prescription

Among neonates exposed to GENT, 90.4% started GENT within the first week; and among neonates exposed to AMK, 77.6% started AMK within the first week.

The mean (±standard deviation) duration of the first treatment was 1.8 (±0.7) for GENT and 1.8 (±0.9) for AMK. It was inversely and significantly related to GA (*p* < 0.0001) for GENT and AMK: 2.1 (±0.9) and 2.2 (±1.1) at 22–26 weeks, 2.0 (±0.8) and 1.9 (±0.9) at 27–31 weeks, 1.8 (±0.7) and 1.7 (±0.7) at 32–36 weeks, and 1.8 (±0.7) and 1.7 (±0.7) at term.

The average of first DD of GENT increased with GA from 2.8 mg/kg/d (±0.5) at 22–26 weeks to 3.2 mg/kg/d (±0.7) at 27–31, 4.2 mg/kg/d (±1.1) at 32–36 weeks, and 4.9 mg/kg/d (±0.6) at >37 weeks (*p* < 0.0001). The average of first DD of AMK increased with GA from 8.9 mg/kg/d (±2.4) at 22–26 weeks to 9.6 mg/kg/d (±2.8) at 27–31, 13.5 mg/kg/d (±4.2) at 32–36 weeks, and 16.2 mg/kg/d (±3.8) at >37 weeks (*p* < 0.0001). The mean of first DD of GENT increased over the study period from 3.9 mg/kg/d (±1.1) in 2017 to 4.4 mg/kg/d (±1.1) in 2020 (*p* < 0.0001). Conversely, mean of first DD of AMK decreased from 13.0 mg/kg/d (±4.2) in 2017 to 12.3 mg/kg/d (±5.1) in 2020 (*p* = 0.001)

The TI between the first 2 doses of GENT was mainly distributed in 3 values during the first week of life: 49.4% at 24 h, 26.4% at 36 h, and 22.9% at 48 h. The corresponding TI for AMK was distributed in 4 values: 48.0% at 24 h, 4.1% at 30 h, 8.5% at 36 h, and 37.1% at 48 h. The predominant TI for GENT treatments was 48 h for 96.8% of the 22–26 weeks and 64.7% of the 27–31 weeks, 36 h for 63.2% of the 32–36 weeks, and 24 h for 93.5% of the term group (Table 1 and Table 2).

Globally, TI is set at 24 h in most GENT (49.4%) and AMK (48.0%) treatments. Afterward, the predominant TI is 36 h for GENT (26.4%) and 48 h for AMK (37.1%).

The increase in postnatal age at first prescription is also associated with a significant decrease in gentamicin TI in very preterm and extremely preterm infants, as shown in Table 3 and Table 4.

The increase in postnatal age at first prescription is also associated with a significant decrease in amikacin TI in very preterm and extremely preterm infants, as shown in Tables S2 and S3 in Supplemental Materials.

#### 3.3.3. The Question of Aminoglycoside over-and under-Dosing

Compared to literature references (Table S1), the rates of neonates exposed to over- and under-dosing were 1.5% and 2.7% for GENT and 0.3% and 1.0% for AMK, respectively. Overdosing rates in GENT-treated neonates decreased significantly over the 4 years of the study: 2.3% in 2017, 1.3% in 2018 and 2019, 1.1% in 2020 (*p* < 0.0001). By GA category, the decrease was significant between 2017 and 2020 at 27–31 weeks (from 4.0% to 1.9%; *p* = 0.04); and also, at 32–36 weeks (from 3.2% to 0.9%; *p* < 0.0001). Likewise, for GENT under-dosing, exposure rates declined from 3.2% in 2017 to 2.3% in 2020 (*p* = 0.02).

Conversely, exposure to AMK over-and under-dosing did not change over the 4-year period regardless of GA category.

**Figure 2:**
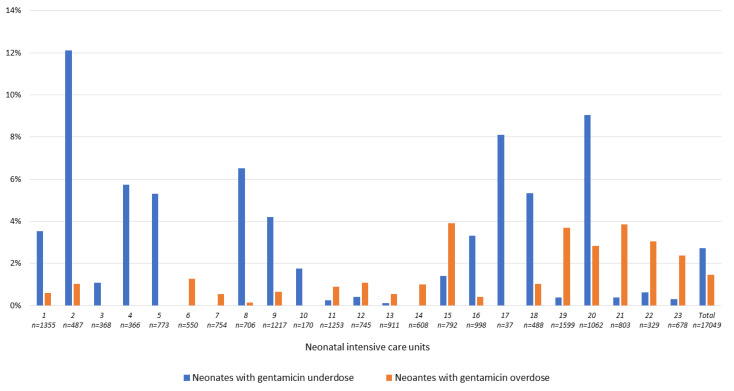
Proportions of neonates with at least one gentamicin over-or under-dosed prescription in 23 neonatal intensive care units.

**Figure 3:**
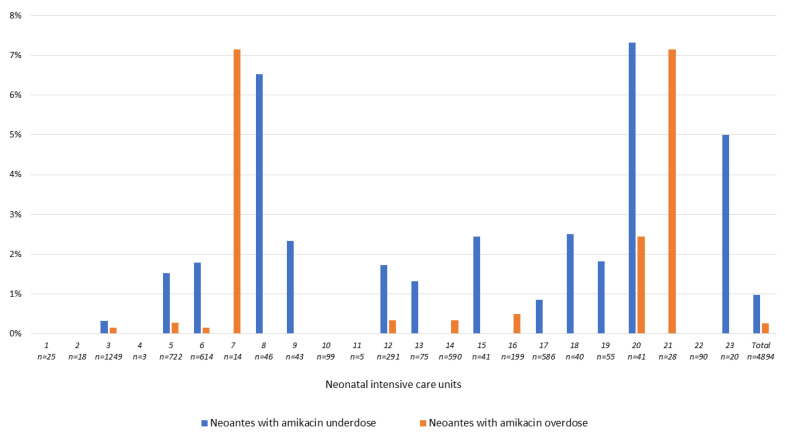
Proportions of neonates with at least one amikacin over-or-under-dosed prescription in 23 neonatal intensive care units.

Variability of over-and underdosing rates in NICU is illustrated in Figure 2 and Figure 3. Depending on hospitals, the overall overdosing rate varied from 0 to 3.9% for GENT (Figure 2) and from 0 to 7.1% for AMK (Figure 3).

Univariate analysis identified several factors associated with GENT overdose (Table 5). In multivariate analysis, the factors significantly associated with GENT overdose were the year of admission, GA below 37 weeks, length of stay, and more than 2 days of administration of the treatment. These factors provide satisfactory precision with an AUC (area under the curve) of 0.75.

## 4. Discussion

This study examined aminoglycosides prescription in 23 NICU that cared for approximately 53,000 neonates over a 4-year period (2017–2020). The global exposure rate was 31.7% for GENT and far below for AMK (9.1%). The CPOE/CDS system allowed a complete prescription of all drugs, a complete record of all prescription data, and an annual benchmarking of antibiotics prescription. In those conditions, we observed a reduction in GENT over-and under-dosing (53% and 28%, respectively) while the corresponding rates for AMK did not vary. Paradoxically, the mean daily dose of GENT increased over the 4-year period. Conversely, the mean daily dose of AMK significantly decreased from 2017 to 2020. Therefore, changes in the daily dose of aminoglycosides were unable to predict changes in over-and under-dosing. In fact, GENT over-dosing was not a uniform phenomenon. It was strongly dependent on GA. It was mainly present at 27–31 weeks and at 32–36 weeks (4.0% and 3.2% in 2017, respectively). Four years later, the incidence of GENT over-dosing in the two categories of preterm neonates fell to 1.9% and 0.9%, respectively. Overall, this population study shows that if the daily dose of aminoglycoside is the cornerstone for benchmarking studies or ASP, additional results (TI, UD, and duration of treatments) are required.

These results about GENT and AMK prescribing are consistent with a recent European survey of antibiotics protocols used in 271 NW [2]. Protocols for antibiotics treatments were related to early-onset neonatal infection, late-onset neonatal infection, and necrotizing enterocolitis. GENT and AMK were the aminoglycosides prescribed in 98% of protocols, the remaining percentage being provided by tobramycin [2] as in this study. More generally, the leading position of GENT is widely recognized, but the rank of AMK varies according to studies. A recent US study by Stark et al. [21, in press] describes a cohort of approximately 800,000 newborns from 363 NICU in the Pediatrix Medical Group; GENT ranks 2nd on the list of all drugs prescribed during 2010–2018, tobramycin ranks 27th, and amikacin ranks 70th [21].

Globally, results from literature and our own research show that neonatologists mostly choose GENT, especially in early-onset neonatal infections [2].

It is interesting to highlight that ASP’s aim is usually to reduce the initiation and duration of antibiotics treatments in NICU by comparing their exposure rates within and between centers. In the year 2015, a huge variability in antibiotics exposure was shown when Schulman et al. [10] reported that exposure to antibiotics among 127 Californian NICU ranged between 2.4% and 97.1%. This rate was independent of proven infection, necrotizing enterocolitis, surgical volume, or mortality. Conversely, an ASP including 89 European NICU from 21 countries found that GENT and AMK were the most frequently prescribed aminoglycoside with little variance in dosing as to the BNFc and Neofax reference values [3]. An Antibiotics Prevalence Survey of Australian hospitalized newborns recently found that the most prescribed antibiotic was also GENT with a median dose of 5 mg/kg/d (IQR: 4; 5) [22]. Even though the benefits of ASP are variously appreciated, a decrease of 34% (16.7 to 12.1%) in antibiotic utilization on the audit day was obtained by the Vermont Oxford Network, which involved 146 NICU participating in an internet-based quality improvement collaborative [23].

Results from this study suggest that the traditional exposure rate to aminoglycosides in NICU is not sufficient to estimate adequacy to professional references. Dosing is a cornerstone to improve the prescription of aminoglycosides. Daily dosing is a useful indicator of good prescribing in adult ICU, and it has also been used in NICU. It is calculated from UD and TI, but it is not, per se, an aim of prescription.

Indeed, the practitioner aims to give an appropriate UD to reach optimal peak and efficacy and an appropriate TI through avoiding toxicity but maintaining efficacy. Blood samples for measurement of peak and through concentrations are useful for close dosing adaptation. However, availability, cost, pain, and blood spoliation limit their use in NICU. Approximately one-third of GENT-treated neonates had blood levels measured in a recent survey [2]. UD and especially TI closely depend on GA and postnatal age. Our study shows how intense they are. Even though the full-term newborns usually necessitated a UD of aminoglycoside given once a day in the first days of life, the TI was enlarged to 36 h or 48 h in infants with the lowest GA and more in some infants. It is important to stress that a TI at 24 h was also the first prescription of GENT in 3.85% of preterm infants below 32 weeks, while the recommendation is 48 h. Therefore, these data indicate that comparison between centers should associate DD, UD, and TI and close analysis of outliers.

It should be noted that prescribing via an identical CPOE/CDS in all the NICUs in this study simplifies assessment prescribing within each unit since a common therapeutic base is initially implemented in each NICU. However, this therapeutic base can be locally modified (UD and TI), which may be an explanation for some inter-unit variations in this study. These variations could also reflect variations observed between the different recommendations of the textbooks and guidelines in the literature [20].

Using a CPOE/CDS system makes prescriptions more secure, as demonstrated in a recent review of all electronic ordering systems with or without cognitive support [24,25,26]. The cognitive support helps the prescriber by automatically providing all parameters of prescription (UD, TI, route, monitoring, preparation, administration). Alerts are required if contraindications or too high or too low doses are prescribed. A drug dictionary is necessary to make prescriptions in accordance with scientific data, even though most drug prescriptions are made off-label. In the near future, new methods based on micro sampling non-invasive samples should overcome actual difficulties for aminoglycosides.

### Limits and Strengths

By nature, this retrospective study suffers limits of which some are specific.

The medication dictionary gives no recommendation about monitoring biological parameters of nephrotoxicity and about blood levels aminoglycoside dosage. It was thought that diversity in local practices and inconsistency in some definitions (i.e., neonatal renal failure) would create a disturbing environment without clear benefit at the benchmarking step.

Variability is high between reference recommendations as well as between centers. This is a strong argument for expanding benchmarking practices and identifying the best practices to obtain optimal efficacy.

## 5. Conclusions

The high quality of prescription in NICU is hard to obtain. Exposure rate to antibiotics has been the first step, but it is not sufficient, particularly for aminoglycosides. Electronic prescription now allows the individualization of prescribing and is also a way for continuous benchmarking at local, regional, or national levels. A transformation of prescription in its quantitative and qualitative aspects will rely on electronic cognitive help.

## Figures and Tables

**Table 1:** Time interval between unit doses at first gentamicin administration by gestational age during the first week of life.

	Gestational Age (Weeks)				
	(22–26) *n* = 1358	(27–31) *n* = 3251	(32–36) *n* = 4171	≥37 *n* = 6627	Total *n* = 15,407
**Time interval, *n* (%)**					
24 h	39 (2.9)	52 (1.6)	1320 (31.6)	6196 (93.5)	7607 (49.4)
36 h	5 (0.4)	1041 (32.0)	2636 (63.2)	393 (5.9)	4075 (26.4)
48 h	1314 (96.8)	2103 (64.7)	83 (2.0)	32 (0.5)	3532 (22.9)

**Table 2:** Time interval between unit doses at first amikacin prescription according to gestational age during the first week of life.

	Gestational Age (Weeks)				
	(22–26) *n* = 449	(27–31) *n* = 1027	(32–36) *n* = 1057	≥37 *n* = 1266	Total *n* = 3799
**Time interval, *n* (%)**					
24 h	1 (0.2)	16 (1.6)	556 (52.6)	1250 (98.7)	1823 (48.0)
30 h	0 (0.0)	37 (3.6)	120 (11.4)	0 (0.0)	157 (4.1)
36 h	2 (0.4)	239 (23.3)	80 (7.6)	1 (0.1)	322 (8.5)
48 h	397 (88.4)	712 (69.3)	297 (28.1)	5 (0.4)	1411 (37.1)
60 h	46 (10.2)	21 (2.0)	0 (0.0)	0 (0.0)	67 (1.8)

**Table 3:** Distribution of time intervals between gentamicin doses in 1511 extremely preterm infants (22–26 weeks) according to postnatal age at first prescription.

	Postnatal Age at First Prescription			
	1st Week *n* = 1358	2nd Week *n* = 81	3rd Week *n* = 29	4th Week *n* = 43
**Time interval, *n* (%)**				
24 h	39 (2.9)	1 (1.2)	1 (3.4)	2 (4.7)
36 h	5 (0.4)	14 (17.3)	8 (27.6)	15 (34.9)
48 h	1314 (96.8)	66 (81.5)	20 (69.0)	26 (60.5)

**Table 4:** Distribution of time intervals between gentamicin doses in 3641 very preterm infants (27–31 weeks) according to postnatal age at first prescription.

	Postnatal Age at First Prescription			
	1st Week *n* = 3251	2nd Week *n* = 228	3rd Week *n* = 99	4th Week *n* = 63
**Time interval, *n* (%)**				
24 h	52 (1.6)	45 (19.7)	25 (25.3)	31 (49.2)
36 h	1041 (32.0)	95 (41.7)	37 (37.4)	16 (25.4)
48 h	2103 (64.7)	84 (36.8)	36 (36.4)	16 (25.4)

**Table 5:** Univariate and multivariate analysis of patients’ characteristics according to gentamicin overdosing.

	No *n* = 16,800	Yes *n* = 249	*p*-Value	OR * (CI 95%)	*p*-Value
**Year of admission, *n* (%)**			<0.0001		<0.0001
2017	3673 (21.9)	87 (34.9)		1	
2018	4448 (26.5)	58 (23.3)		0.55 (0.39; 0.77)	
2019	4409 (26.2)	56 (22.5)		0.54 (0.38; 0.76)	
2020	4270 (25.4)	48 (19.3)		0.47 (0.33; 0.68)	
**Male, *n* (%)**	9706 (57.8)	141 (56.6)	0.72		
**Birth weight (g), mean (±SD)**	2366 (1053)	1688 (804)	<0.0001		
**Birth weight Z-score, mean (±SD)**	−0.2 (1.0)	−0.2 (0.9)	0.61		
**Intrauterine growth restriction (birth weight z-score < −1.28), *n* (%)**	2159 (12.9)	23 (9.2)	0.09		
**Gestational age (weeks), *n* (%)**			<0.0001		<0.0001
(22; 26)	1484 (8.8)	27 (10.8)		1.45 (0.82; 2.56)	
(27; 31)	3535 (21.0)	106 (42.6)		3.02 (1.93; 4.73)	
(32; 36)	4394 (26.2)	81 (32.5)		2.60 (1.71; 3.97)	
≥37	7387 (44.0)	35 (14.1)		1	
**Length of stay (days), mean (±SD)**	25.6 (33.5)	42.9 (37.1)	<0.0001		
**Length of stay (days), *n* (%)**					<0.0001
(1; 4)	4303 (25.6)	14 (5.6)		1	
(5; 11)	4235 (25.2)	32 (12.9)		1.87 (0.99; 3.54)	
(12; 35)	4215 (25.1)	91 (36.5)		3.65 (2.02; 6.62)	
≥36	4047 (24.1)	112 (45.0)		3.30 (1.76; 6.19)	
**Amikacin exposure during hospitalization, *n* (%)**	707 (4.2)	14 (5.6)	0.27		
**Neonates with at least one amikacin underdose, *n* (%)**	12 (0.1)	0 (0.0)	0.99		
**Duration of gentamicin (days), *n* (%)**			<0.0001		<0.0001
(1; 2)	13,498 (80.3)	150 (60.2)		1	
(3; 4)	2627 (15.6)	55 (22.1)		1.40 (1.01; 1.94)	
≥5	675 (4.0)	44 (17.7)		4.08 (2.77; 6.03)	
**Postnatal age at first prescription of gentamicin, mean (±SD)**	1.9 (4.6)	3.0 (6.3)	0.07		

OR: Odds ratio; CI: Confidence interval; * adjusted on year of admission, gestational age, length of stay, and duration of gentamicin.

## Data Availability

The raw data supporting the conclusions of this article will be made available by the authors without undue reservation.

## Supplementary Materials

The following are available online at https://www.mdpi.com/article/10.3390/antibiotics10111422/s1, Table S1. Unit daily doses of gentamicin and amikacin, according to the literature. Table S2. Distribution of time intervals between amikacin doses in 680 extremely preterm infants (22–26 weeks) according to postnatal age at first prescription. Table S3. Distribution of time intervals between amikacin doses in 1366 very preterm infants (27–31 weeks) according to postnatal age at first prescription.
