# Supplementary material for: Prescription of Aminoglycosides in 23 French Neonatal Intensive Care Units
Source: Antibiotics (Basel). 2021 Nov 20;10(11):1422. doi: 10.3390/antibiotics10111422 (PMC8614949; doi:10.3390/antibiotics10111422)
Supplement: Supplementary file 1 [file antibiotics-10-01422-s001.zip › antibiotics-1452803-supplementary.pdf]

Supplementary Materials:

Table S1. Unit daily doses of gentamicin and amikacin according to the literature

Source	GA* (weeks)	PMA* (weeks)	PNA* (days)	Weigth (kg)	Recommendations	Minimum unit daily dose (mg/kg)	Maximum unit daily dose (mg/kg)
**Gentamicin**							
Handbook (Bradley et Redbook 2021)	< 30		≤ 14		5mg/kg/dose every 48h	2,5	2,5
	< 30		≥ 15		5mg/kg/dose every 36h	3,333	3,333
	30-34		≤ 10		5mg/kg/dose every 36h	3,333	3,333
	30-34		11-60		5mg/kg/dose every 24h	5	5
	≥ 35		≤ 7		4mg/kg/dose every 24h	4	4
	≥ 35		8-60		5mg/kg/dose every 24h	5	5
Handbook (Bradley 2015)	< 32		≤ 14		5mg/kg/dose every 48h	2,5	2,5
	< 32		≥ 15		5mg/kg/dose every 36h	3,333	3,333
	32-36		≤ 7		4mg/kg/dose every 36h	2,667	2,667
	32-36		8-60		4mg/kg/dose every 24h	4	4
	≥ 37		≤ 28		4mg/kg/dose every 24h	4	4
Handbook (Redbook 2015)			≤ 14	< 1	5mg/kg/dose every 48h	2,5	2,5
			15-28	< 1	5mg/kg/dose every 36h	3,333	3,333
			≤ 7	1-2	5mg/kg/dose every 48h	2,5	2,5
			8-28	1-2	5mg/kg/dose every 36h	3,333	3,333
			≤ 7	> 2	4mg/kg/dose every 24h	4	4
			8-28	> 2	4-5mg/kg/dose every 24h	4	5
ANSM (Afssaps)		< 30			7 mg/kg every 48h	3,5	3,5
		30-33			6,5 mg/kg every 36h	4,333	4,333
		34-36			6 mg/kg every 24-36h	4	6
		≥ 37			5,5 mg/kg every 24h	5,5	5,5
SFN	34 - 36				6mg/kg/24h	6	6
	≥ 37				5mg/kg/24h	5	5
BNFc	≥ 37		<7		5 mg/kg every 36h	3,333	3,333
	≥ 37		7-28		5 mg/kg every 24h	5	5
**Amikacin**							
Handbook (Bradley 2016)	< 30		<14		15mg/kg/dose every 48h	7,5	7,5
	< 30		≥15		15mg/kg/dose every 36h	10	10
	30-34		≤ 60		15mg/kg/dose every 24h	15	15
	≥35		≤7		15mg/kg/dose every 24h	15	15
	≥35		≥8		17,5mg/kg/dose every 24h	17,5	17,5
Handbook (Redbook 2015-2021)			≤14	<1	15 mg/kg/dose every 48h	7,5	7,5
			15-28	<1	15 mg/kg/dose every 24h	15	15
			≤7	1-2	15 mg/kg/dose every 48h	7,5	7,5

Source	GA* (weeks)	PMA* (weeks)	PNA* (days)	Weigth (kg)	Recommendations	Minimum unit daily dose (mg/kg)	Maximum unit daily dose (mg/kg)
			8-28	1-2	15 mg/kg/dose every 24h	15	15
			≤7	>2	15 mg/kg/dose every 24h	15	15
			8-28	>2	17,5 mg/kg/dose every 24h	17,5	17,5
Handbook (multiples articles)		≤ 27			15-20mg/kg/dose every 48h	7,5	10
		28-33			15-20mg/kg/dose every 36h	10	13,333
		≥34			15-20mg/kg/dose every 24h	15	20
Handbook (Redbook 2012)			≤14	<1	15 mg/kg/dose every 48h	7,5	7,5
			15-28	<1	15 mg/kg/dose every 24-48h	7,5	15
			≤7	1-2	15 mg/kg/dose every 48h	7,5	7,5
			8-28	1-2	15 mg/kg/dose every 24-48h	7,5	15
			≤7	>2	15 mg/kg/dose every 24h	15	15
			8-28	>2	15 mg/kg/dose every 12-24h	15	30
Handbook			≤7	≥2	15-20 mg/kg/day divided every 12h	15	20
			> 7	≥2	30 mg/kg/day divided every 8h	30	30
ANSM (Afssaps)		< 30			35 mg/kg every 60h	14	14
		30-33			32,5 mg/kg every 48h	16,25	16,25
		34-36			30 mg/kg toutes 36h	20	20
		≥ 37			27,5 mg/kg every 24 à 36h	18,333	27,5
BNFc	≥ 37				15 mg/kg every 24 hours, intravenous injection to be administered over 3–5 minutes	15	15
	≥ 37				Loading dose 10 mg/kg	10	10
	≥ 37				then 7.5 mg/kg every 12 hours, intravenous injection to be administered over 3–5 minutes.	15	15

* GA, gestational age; PMA, postmenstrual age; PNA, postnatal age.

Table S2. Distribution of time intervals between amikacin doses in 680 extremely preterm infants (22-26 weeks) according to postnatal age at first prescription

	Postnatal age at first prescription			
	**1st week n=449**	**2nd week n=126**	**3rd week n=69**	**4th week n=36**
**Time Intervals, n (%)**				
24 h	1 (0.2)	1 (0.8)	2 (2.9)	5 (13.9)
30 h	0 (0.0)	0 (0.0)	0 (0.0)	0 (0.0)
36 h	2 (0.4)	12 (9.5)	26 (37.7)	22 (61.1)
48 h	397 (88.4)	90 (71.4)	19 (27.5)	2 (5.6)
60 h	46 (10.2)	19 (15.1)	19 (27.5)	7 (19.4)

Table S3. Distribution of time intervals between amikacin doses in 1366 very preterm infants (27-31 weeks) according to postnatal age at first prescription

	**Postnatal age at first prescription**			
	**1st week n=1,027**	**2nd week n=206**	**3rd week n=83**	**4th week n=50**
**Time Intervals, n (%)**				
24 h	16 (1.6)	10 (4.9)	9 (10.8)	9 (18.0)
30 h	37 (3.6)	9 (4.4)	5 (6.0)	1 (2.0)
36 h	239 (23.3)	123 (59.7)	46 (55.4)	30 (60.0)
48 h	712 (69.3)	54 (26.2)	22 (26.5)	10 (20.0)
60 h	21 (2.0)	8 (3.9)	1 (1.2)	0 (0.0)
